# Predicting IDH and 1p/19q molecular status of gliomas with multi-b values DWI

**DOI:** 10.3389/fonc.2025.1551023

**Published:** 2025-07-30

**Authors:** Shanshan Zhao, Peipei Wang, Eryuan Gao, Mengzhu Wang, Guang Yang, Shouhui Niu, Mengjiao Pan, Kai Zhao, Jingliang Cheng, Xiaoyue Ma

**Affiliations:** ^1^ Department of Magnetic Resonance Imaging, The First Affiliated Hospital of Zhengzhou University, Zhengzhou, China; ^2^ MR Research Collaboration, Siemens Healthineers Ltd., Beijing, China; ^3^ Shanghai Key Laboratory of Magnetic Resonance, East China Normal University, Shanghai, China; ^4^ Department of Medical Imaging, Shenqiu County People’s Hospital, Zhoukou, China

**Keywords:** diffusion magnetic resonance imaging, continuous time random walk, glioma, IDH, 1p/19q

## Abstract

**Background and purpose:**

In the 2021 WHO Classification, the importance of molecular pathology in glioma diagnosis has been emphasized, particularly the status of isocitrate dehydrogenase (IDH) mutation and 1p/19q co-deletion. Advanced magnetic resonance diffusion-weighted imaging (DWI) including mono-exponential (Mono), intravoxel incoherent motion (IVIM), stretched exponential model (SEM) techniques are beneficial for non-invasive prediction of these molecular markers. The continuous-time random walk (CTRW) model mitigates the empirical nature of the SEM and has shown promising results in grading gliomas. However, the application of CTRW model in prediction of IDH and 1p/19q molecular phenotypes in adult diffuse gliomas remains underreported. This study compares the clinical utility of mono-exponential, IVIM, SEM, and CTRW models for predicting IDH and 1p/19q molecular status in adult diffuse gliomas.

**Materials and methods:**

Data of adult diffuse glioma patients from January 2021 to August 2023 were collected. The multi-b-value DWI was acquired using a spin-echo echo-planar imaging sequence with 13 b-values (0, 10, 20, 30, 50, 70, 100, 150, 200, 400, 800, 1500, 2000 s/mm²) in 30 diffusion-encoding directions. Multi-b-value DWI images were post-processed to generate parametric maps based on the mono-exponential (Mono), the intravoxel incoherent motion (IVIM), the stretched exponential model (SEM) and the continuous-time random walk (CTRW) models. The mean parameter values of solid tumor regions were calculated. An independent sample *t*-test or Mann-Whitney *U* test was used for comparisons between different subtypes of glioma. Receiver operating characteristic (ROC) analyses were used to assess diagnostic performance.

**Results:**

A total of 95 glioma patients were included in the study. For predicting IDH status, CTRW_α exhibited the largest effect size and best diagnostic performance with an AUC of 0.761. At a threshold of 0.855, the sensitivity was 0.651, the specificity was 0.846, and the accuracy was 0.758. In predicting 1p/19q status in IDH-mutant gliomas, CTRW_α again showed the largest effect size and the best diagnostic performance with an AUC of 0.790. At a threshold of 0.886, sensitivity was 0.750, specificity was 0.903, and accuracy was 0.860.

**Conclusions:**

The CTRW model could help predict IDH and 1p/19q status in adult diffuse gliomas.

## 1 Introduction

Diffuse gliomas are the most common primary malignant brain tumors in adults (1). In the 2021 fifth edition of the WHO’s Central Nervous System tumor classification, a new grading system is applied within each subtype, replacing the previous approach that assigned a general grade across all subtypes (2). This updated grading system comprehensively considers the responses to clinical treatments of different glioma subtypes (3), establishing a closer link with the clinical prognosis of gliomas (4). Two key genetic markers, isocitrate dehydrogenase (IDH) and chromosome 1p/19q, are critical in guiding treatment and prognosis (5). IDH catalyzes the oxidative decarboxylation of isocitrate to α-ketoglutarate (α-KG), thereby contributing to cellular defense against oxidative stress (6). IDH gene mutations are present in approximately 50%–80% of WHO grade 2 and 3 gliomas, with IDH1 mutations being more prevalent than IDH2 mutations (7, 8). The mutant IDH1 enzyme acquires a neomorphic activity that converts α-KG into the oncometabolite 2-hydroxyglutarate (2-HG) (9), which competitively inhibits α-KG–dependent dioxygenases involved in DNA repair and epigenetic regulation (10). Patients with IDH1-mutant gliomas have significantly better prognosis compared to those with wild-type IDH1, and IDH mutation status has been recognized as an independent prognostic marker (2, 11). The 1p/19q codeletion, resulting from an unbalanced translocation between chromosomes 1p and 19q, is a hallmark of oligodendrogliomas (12, 13). Tumors harboring this codeletion demonstrate increased sensitivity to radiochemotherapy and are associated with prolonged survival compared to those without the deletion (3). In recent years, the emergence of targeted and immunotherapeutic approaches has opened new avenues for the clinical management of gliomas (14–16). Accurate and reliable molecular diagnosis is not only integral to the WHO classification of gliomas but also critical for identifying actionable therapeutic targets, guiding individualized treatment decisions, and prognosticating outcomes. Currently, the molecular genetic diagnosis of gliomas relies on pathological examination of tissue samples (17) but this invasive method carries risks (18). A simple, accurate, and non-invasive diagnostic method could benefit patients unable to undergo surgery and provide essential information for advanced surgical alternatives (14–16).

Magnetic resonance imaging (MRI) is the primary imaging diagnostic tool in evaluating glioma patients. Increasing attention has been given to functional MRI methods for non-invasive molecular diagnosis and grading prediction in gliomas before surgery (19–21).In IDH-wildtype gliomas, invasive growth and rapid proliferation often lead to insufficient blood supply and central necrosis, typically presenting as ill-defined margins and ring-like enhancement. In contrast, gliomas lacking enhancement and exhibiting well-defined, sharp margins are more commonly IDH-mutant (22). The excellent performance of conventional MRI combined with radiomics and deep learning in predicting glioma molecular information has been well demonstrated (23–25). However, these artificial intelligence techniques still face challenges such as the black-box effect and limited interpretability. Functional MRI provides quantitative or semi-quantitative parameters related to the pathophysiological characteristics of tumors. Perfusion imaging can characterize tumor hemodynamic differences, with IDH-wildtype gliomas exhibiting a more disrupted blood-brain barrier and higher vascular permeability, while gliomas with 1p/19q co-deletion often show higher perfusion levels (26). Advanced spectroscopic MRI predicts IDH status by detecting the mutant IDH product, 2-hydroxyglutaric acid (2HG) (27). Diffusion-weighted imaging (DWI) generates parameter maps for the quantitative evaluation of tissue microstructure and so serve as tools for determining the molecular characteristics of gliomas.

In current clinical applications, DWI typically uses two sets of images with b-values of 0 s/mm² and 1000 s/mm², and the mono-exponential (Mono) model based on the Gaussian distribution is applied during post-processing to calculate the apparent diffusion coefficient (ADC), which assesses tumor cell density. However, water molecule diffusion may deviate from a Gaussian pattern, leading to inaccuracies in ADC measurements in highly heterogeneous tumor tissues. Furthermore, blood flow signals within the tumor can interfere with these measurements (28). To address this, the intravoxel incoherent motion (IVIM) based on a bi-exponential model has been introduced (29). IVIM separates water molecule diffusion into fast and slow components, differentiating diffusion and microcirculation perfusion. IVIM parameters include: perfusion fraction (*f*), indicating the vascular volume fraction within a voxel; fast diffusion coefficient (D^*^), representing water movement within the microvascular; and slow diffusion coefficient (D), reflecting tissue water diffusion.

While the IVIM improves diffusion analysis, some argue that separating water movement into two components is overly simplistic, with the stretched exponential model (SEM) (30) being developed to address this. In SEM, the decay of the diffusion-weighted signal is described as a continuous distribution rather than limited to specific sources. This model introduces the distributed diffusion coefficient (DDC) to evaluate the diffusion rate and the parameter α to quantify heterogeneity. However, SEM_α is empirical and its fitting process relies on experimental data, lacking comprehensive theoretical support (31). The development of the continuous-time random walk (CTRW) model mitigates the empirical nature of SEM (32). In contrast to the SEM, which condenses all intravoxel heterogeneity into a single stretching exponent (α) and a mean diffusivity (DDC), the CTRW model-based on the random walk theory-characterizes diffusion heterogeneity in two independent and complementary dimensions: space and time (33). The time heterogeneity index α, describes the likelihood of water molecules being trapped or released during diffusion through complex tissues (e.g., cell–matrix adhesion and slowed subdiffusion) (34). As a complement, β is related to spatial diffusion heterogeneity (35), quantifying the variability in water molecule movement distances (e.g., perivascular corridor-mediated long-range migration) (33). This dual-exponent formalism not only provides direct mechanistic links between DWI signal attenuation and specific microstructural features (adhesion kinetics vs. structural conduits) but also inherently spans diffusion regimes from subdiffusion (α<1, β≈2) to Lévy‐flight superdiffusion (β<2). In contrast, SEM’s single-parameter description cannot distinguish whether non-monoexponential decay arises from prolonged local retention or sporadic long jumps. CTRW model also calculates the anomalous diffusion coefficient (D_m_) to quantify tissue cell density (36).D_m_ shares a similar physiological interpretation with the conventional ADC (36), and enables the CTRW model to be used with the conventional water diffusion information preserved, which can be applied to characterize tissue cellularity and ischemic changes (37). Recent studies have shown growing interest in CTRW for noninvasive preoperative evaluation of breast cancer (35, 38). Mao et al. (39) compared the performance of mono-exponential, IVIM, fractional order calculus (FROC), and CTRW models in predicting HER2 expression levels, demonstrating the superior diagnostic efficacy of CTRW. In central nervous system tumors, CTRW has been successfully applied to predict tumor grading in pediatric brain tumors (33, 40) and preoperative glioma stratification (36, 41). Karaman et al. (33) found significantly lower α, β, and D_m_ values in high-grade pediatric brain tumors compared to low-grade ones, likely due to increased microstructural complexity from hemorrhage, necrosis, vasogenic edema, angiogenesis, and higher cellular proliferation. CTRW parameters (α, β, D_m_) were also shown to decrease with increasing tumor grade (36). These findings highlight the model’s ability to capture microenvironmental heterogeneity-particularly valuable in brain tumors where necrosis, cystic degeneration, hemorrhage, edema, calcification, and diverse cellular subtypes are commonly present. Compared to mono-exponential diffusion models, CTRW provides enhanced sensitivity to such heterogeneity, offering a promising tool for more accurate tumor characterization.

Previous studies (28, 33, 36, 42, 43) have shown that the mono-exponential, IVIM, and SEM models effectively predict glioma grading, IDH, and 1p/19q status. However, the use of the CTRW model for predicting genetic status in gliomas is less explored. This study evaluates and compares the clinical potential of the mono-exponential, IVIM, SEM, and CTRW models in predicting IDH and 1p/19q molecular status in adult diffuse gliomas.

## 2 Material and methods

### 2.1 Study participants

This study was approved by the Medical Ethics Review Committee of the First Affiliated Hospital of Zhengzhou University (Approval Number: 2019-KY-231). Written informed consent was waived. We prospectively collected data from patients diagnosed with adult diffuse glioma at our institution between January 2021 and August 2023. Inclusion criteria were: 1. MRI with a multi-b-value DWI sequence performed in two weeks before surgery; 2. postoperative diagnosis of adult diffuse glioma based on the 2021 WHO CNS tumor classification. Exclusion criteria were: 1. prior anti-tumor treatments before MRI; 2. incomplete molecular data from glioma pathology; 3. low MRI quality due to severe susceptibility or motion artifacts.

### 2.2 Molecular studies

IDH genes were detected using Sanger sequencing. The presence of either IDH1 R132H or IDH2 R172H mutation was considered diagnostic of an IDH mutation. Chromosome 1p/19q status was assessed using fluorescence *in situ* hybridization (FISH).

### 2.3 MRI protocol

All scans were performed using a Siemens 3.0T MRI scanner (MAGNETOM Prisma, Siemens Healthcare, Erlangen, Germany) with a 64-channel head and neck coil. The conventional MRI sequences included: axial T1-weighted imaging (T1WI); axial T2-weighted imaging; axial T2-weighted dark-fluid imaging; and delayed-enhancement 3D-T1 magnetization-prepared rapid gradient echo (MPRAGE) sequence.

The multi-b-value DWI was acquired using a spin-echo echo-planar imaging sequence: TE = 71.0 ms, TR = 2500.0 ms, 60 slices, slice thickness = 2.2 mm, b-values = 0, 10, 20, 30, 50, 70, 100, 150, 200, 400, 800, 1500, 2000 s/mm², 30 diffusion-encoding directions, FOV = 220 mm × 220 mm, matrix = 100 × 100, scan time = 2 minutes 48 seconds, total scan time = 11 minutes 15 seconds.

### 2.4 Image processing and analysis

The raw DICOM data of multi-b-value DWI images was transformed to the NIfTI1.1 format with the MRIcron (https://www.nitrc.org/projects/mricron). All DWI data underwent eddy current and motion correction using the Diffusionkit tool (44). Post-processing of multi-b-value DWI images was performed using an in-house postprocessing software (BoDiLab) based on the open-resource tool DIPY (Diffusion Imaging in Python, https://dipy.org) (34, 45) to generate parametric maps from various DWI models. S(0) and S(b) represent the signal intensities at b = 0 and non-zero b-values, respectively.

Based on the mono-exponential model, ADC map was generated:


$$\frac{S(b)}{S(0)}=\exp\left(-b\cdot ADC\right)$$

Based on the IVIM model, the D, D^*^, and *f* maps were generated:


$$\frac{S(\begin{array}{c} b \end{array})}{S(\begin{array}{c} 0 \end{array})}=\left[\begin{array}{c} f\cdot \exp(\begin{array}{c} -b\cdot {\text{D}}^{*} \end{array}) \end{array}\right]+\left[\begin{array}{c} \left(\begin{array}{c} 1-f \end{array})\cdot \exp(\begin{array}{c} -b\cdot \text{D} \end{array}\right) \end{array}\right]$$

Based on the SEM, DDC and the heterogeneity index (α) were generated:


$$\frac{S(b)}{S(0)}=\exp\left[-{\left(\begin{array}{c} b\cdot \text{DDC} \end{array}\right)}^{\text{α}}\right]$$

Based on the CTRW model, D_m_, the time diffusion heterogeneity index (α), and the spatial diffusion heterogeneity index (β) were generated, and the β parameter is restricted to a maximum value of 1 through normalization:


$$\frac{S(b)}{S(0)}=E_{\alpha }\left[\begin{array}{c} -{\left(b\cdot {\text{D}}_{\text{m}}\right)}^{\beta } \end{array}\right]$$

**Figure 1:**
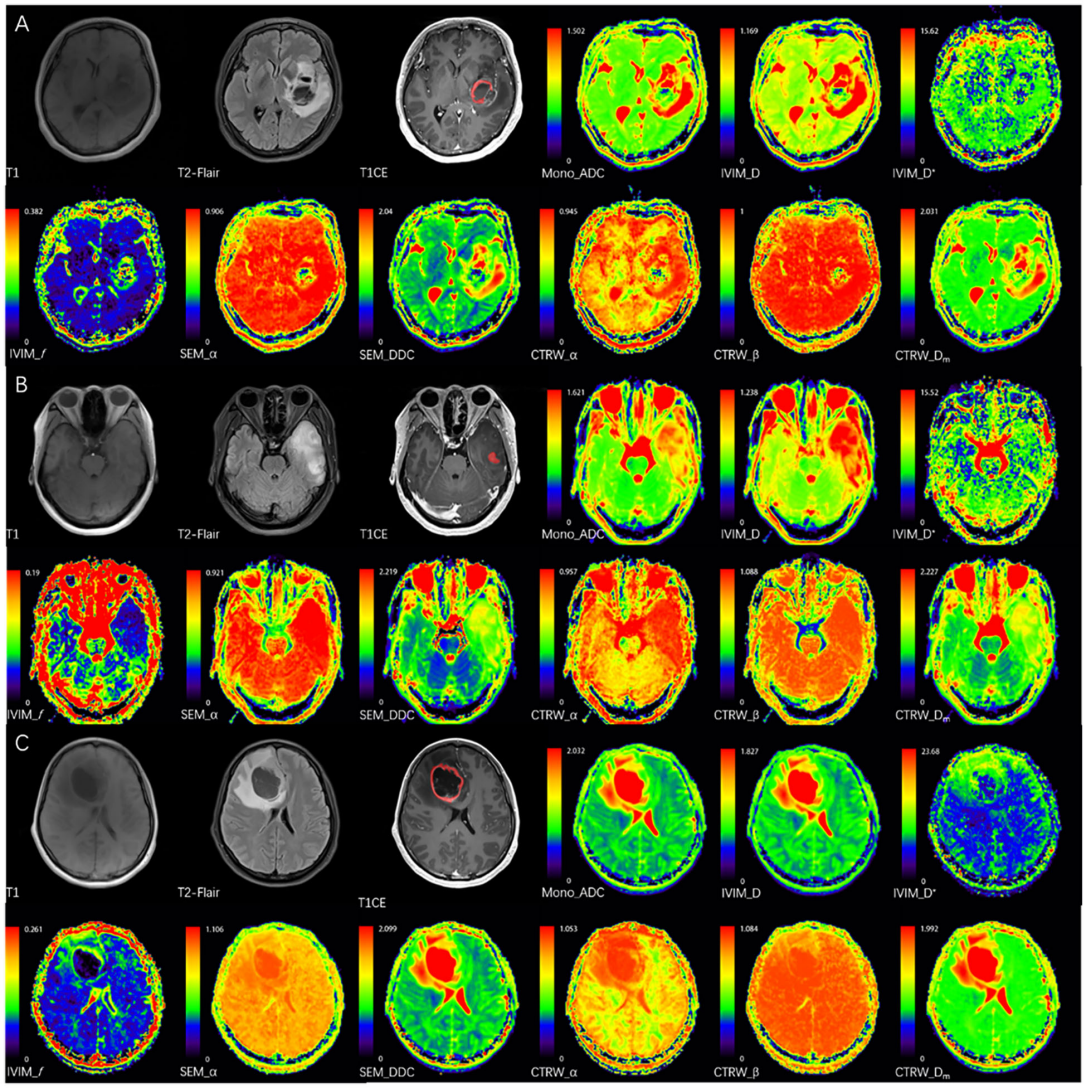
The red areas represent the delineated VOIs. **(A)** A 52-year-old female with IDH wild-type glioblastoma in the left basal ganglia, CTRW_α = 0.714, below the IDH threshold (0.855), correctly classified by imaging analysis. **(B)** A 38-year-old female with IDH-mutant & 1p/19q co-deletion oligodendroglioma in the left temporal lobe, CTRW_α = 0.872, above the IDH threshold and below the 1p/19q threshold (0.886), correctly classified by imaging analysis. **(C)** A 38-year-old female with IDH-mutant astrocytoma without 1p/19q co-deletion in the right frontal lobe, CTRW_α = 0.916, above the IDH or 1p/19q thresholds, correctly classified by imaging analysis.

ITK-SNAP software (http://www.itksnap.org/pmwiki/pmwiki.php) was used to register each patient’s diffusion parameter maps to axial T2 dark-fluid and enhanced 3D-T1 MPRAGE images. A senior physician with 3 years of experience manually delineated the solid tumor regions while avoiding areas of hemorrhage, calcification, edema, necrosis, or cystic changes to define volumes of interest (VOIs), and the MRI morphological characteristics of the tumor (location (46), presence of hemorrhage, cystic/necrosis, edema, and enhancement) were assessed. The delineations and the morphological characteristics were reviewed by an associate chief physician with 11 years of experience. Both of the physicians were blinded to the final molecular diagnosis. The solid tumor region was defined as the enhanced region on enhanced 3D-T1 MPRAGE images, or, if no enhancement was present, as the hyperintense area on T2 dark-fluid sequence (**Figure 1**). DWI parameter maps and VOI files were imported into FAE software (https://github.com/salan668/FAE) (47) to calculate the mean values of each parameter within the VOIs.

### 2.5 Statistical analysis

Statistical analysis was performed with R software (version 4.3.1; https://www.R-project.org/). The interobserver and concordance of DWI values was assessed using intraclass correlation coefficient (ICC) analysis with a two-way random-effects model. Continuous variables were assessed for normality using the Shapiro-Wilk test and for homogeneity of variance using Levene’s test. For normally distributed data, independent samples t-tests or Welch’s t-tests were used, depending on variance homogeneity. Non-normally distributed data were analyzed with the Mann-Whitney U test. For each DWI parameter, we independently tested its association with two molecular markers (IDH and 1p/19q), resulting in a total of 9 parameters × 2 genes = 18 hypotheses. Benjamini-Hochberg correction was applied to adjust these 18 P values of parameters for multiple comparisons using the function: p.adjust(). Results are reported as ‘mean ± standard deviation’ or ‘median (25th percentile, 75th percentile)’. Effect sizes were calculated with Cohen’s d values, where an absolute value greater than 0.8 indicates a strong effect. Categorical variables were analyzed using the chi-square test. Receiver operating characteristic (ROC) curves assessed the performance of diffusion parameters in predicting IDH and 1p/19q status, with diagnostic performance quantified by the area under the curve (AUC). The optimal threshold was determined using the Youden index, and sensitivity, specificity, and accuracy were calculated. Statistical significance was set at *P* < 0.05.

## 3 Results

### 3.1 Patients characteristics

This study included 95 glioma patients (54 males, 41 females) aged 24 to 70 years, with a mean age of 48. According to the 2021 WHO CNS tumor classification, the tumors were categorized as: IDH wild-type gliomas (glioblastomas, 52 cases), IDH-mutant without 1p/19q codeleted gliomas (astrocytomas, 12 cases), and IDH-mutant with 1p/19q codeleted gliomas (oligodendrogliomas, 31 cases). Patients with IDH wild-type gliomas were significantly older than those with IDH-mutant gliomas (*P* < 0.001). No significant age differences were found between IDH-mutant gliomas with and without 1p/19q co-deletion. Gender distribution did not significantly differ among glioma subtypes. In terms of MRI morphology, IDH wild-type gliomas are significantly more likely to exhibit cystic/necrotic changes, edema, and contrast enhancement, whereas IDH mutant gliomas are predominantly located in the frontal lobe or insular lobe. **Table 1** summarizes the patients’ demographic characteristics and the morphological characteristics.

**Table 1:** Patient characteristics.

Parameter	IDH wild-type	IDH mutant	*P* (IDH)	IDH mutant & 1p/19q codeleted	IDH mutant & 1p/19q non-codeleted	*P* (1p/19q)
Age	54 (50, 60)	41 (34, 49)	<0.001	42 ± 9	38 ± 9	0.225
Gender (M/F)	28/24	26/17	0.518	20/11	6/6	0.599
Cystic/Necrosis (Y/N)	44/8	28/15	0.027	21/10	7/5	0.822
Hemorrhage (Y/N)	4/48	0/43	0.124	0/31	0/12	–
Edema (Y/N)	42/10	24/19	0.009	17/14	7/5	0.836
Enhancement (Y/N)	47/5	18/25	<0.001	13/18	5/7	0.987
VOI volume (mm^2^)	10225 (5802.5,17465)	15300 (2907.5,40655)	0.134	13060 (2609.5,31160)	28930 (9138.5,88145)	0.142
Location						
-Frontal or Insula (Y/N)	18/34	31/12	<0.001	22/9	9/3	>0.99
-Basal ganglia or Thalamus (Y/N)	6/46	1/42	0.188	1/30	0/12	>0.99
-Other (Y/N)	28/24	12/31	0.011	9/22	3/9	>0.99

IDH, isocitrate dehydrogenase; M, Male; F, Female; Y, Yes; N, No; VOI, volume of interest.

### 3.2 DWI parameters in predicting IDH status

The interobserver and reproducibility were good for all DWI parameters (ICC = 0.918–0.996). The ICCs for different DWI values are provided in **Supplementary Table S1**.

**Figure 2:**
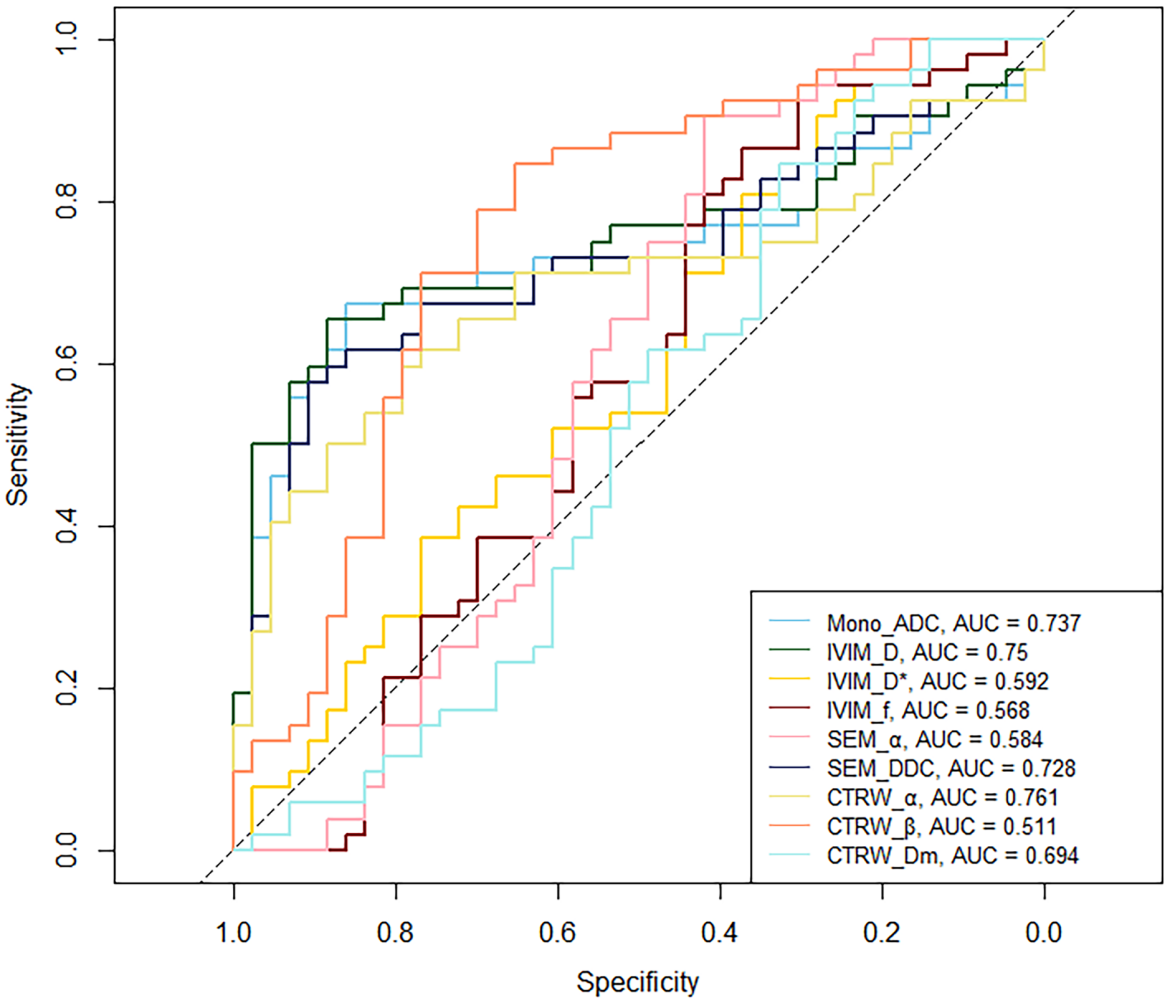
ROC curve of IDH status predicting by DWI parameters.

Mono_ADC, IVIM_D, SEM_DDC, CTRW_α, and CTRW_D_m_ were significantly higher in IDH-mutant gliomas compared to IDH wild-type gliomas (*P* < 0.05). No significant differences were observed in other diffusion parameters between the two groups (*P* > 0.05) (**Table 2**). CTRW_α had the largest effect size for predicting IDH genotype (Cohen’s d = -0.897), as shown in **Table 2**. ROC analysis (**Table 3**) indicated that CTRW_α provided the best diagnostic performance for predicting IDH genotype (AUC = 0.761). At a threshold of 0.855, sensitivity was 0.651, specificity was 0.846, and accuracy was 0.758 (**Figure 2**).

**Table 2:** Parameter values between different IDH status.

Parameter	IDH wild-type	IDH mutant	*t*/*U*	*P*	Cohen’s d
Mono_ADC	1.088(0.997,1.219)	1.200(1.141,1.307)	1648	< 0.001	-0.704
IVIM_D	0.983(0.883,1.083)	1.099(1.028,1.157)	1677	< 0.001	-0.769
IVIM_D^*^	7.298(6.630,7.929)	7.443(6.991,8.676)	1324	0.204	-0.424
IVIM_*f*	0.089(0.075,0.106)	0.085(0.060,0.104)	967	0.363	0.149
SEM_α	0.867(0.840,0.88)	0.875(0.835,0.911)	1306	0.242	-0.123
SEM_DDC	1.117(1.029,1.274)	1.233(1.163,1.342)	1628	< 0.001	-0.666
CTRW_α	0.825(0.789,0.849)	0.867(0.844,0.890)	1702	< 0.001	-0.897
CTRW_β	0.938(0.917,0.956)	0.935(0.904,0.968)	1093	0.906	0.202
CTRW_D_m_	1.218(1.102,1.365)	1.335(1.254,1.396)	1551	0.004	-0.599

IDH, isocitrate dehydrogenase; ADC, apparent diffusion coefficient; IVIM, intravoxel incoherent motion; D, slow diffusion coefficient; D^*^, fast diffusion coefficient; SEM, stretched exponential model; DDC, distributed diffusion coefficient; CTRW, continuous-time random walk; D_m_, anomalous diffusion coefficient.

**Table 3:** ROC curve analysis of parameters for predicting IDH status.

Parameter	Threshold	*P*	AUC (95% *CI*)	Sensitivity	Specificity	Accuracy
Mono_ADC	1.122	< 0.001	0.737 (0.634,0.839)	0.860	0.673	0.758
IVIM_D	0.998	< 0.001	0.750 (0.644,0.843)	0.884	0.654	0.758
IVIM_D^*^	8.586	0.061	0.592 (0.470,0.707)	0.279	0.904	0.621
IVIM_*f*	0.067	0.140	0.568 (0.447,0.686)	0.372	0.865	0.642
SEM_α	0.896	0.093	0.584 (0.456,0.703)	0.419	0.904	0.684
SEM_DDC	1.130	< 0.001	0.728 (0.623,0.826)	0.907	0.577	0.726
CTRW_α	0.855	< 0.001	0.761 (0.661,0.854)	0.651	0.846	0.758
CTRW_β	0.909	0.430	0.511 (0.388,0.634)	0.326	0.846	0.611
CTRW_D_m_	1.248	< 0.001	0.694 (0.585,0.793)	0.791	0.596	0.684

AUC, area under the curve; CI, confidence interval; ADC, apparent diffusion coefficient; IVIM, intravoxel incoherent motion; D, slow diffusion coefficient; D^*^, fast diffusion coefficient; SEM, stretched exponential model; DDC, distributed diffusion coefficient; CTRW, continuous-time random walk; D_m_, anomalous diffusion coefficient.

### 3.3 DWI parameters in predicting 1p/19q status

**Figure 3:**
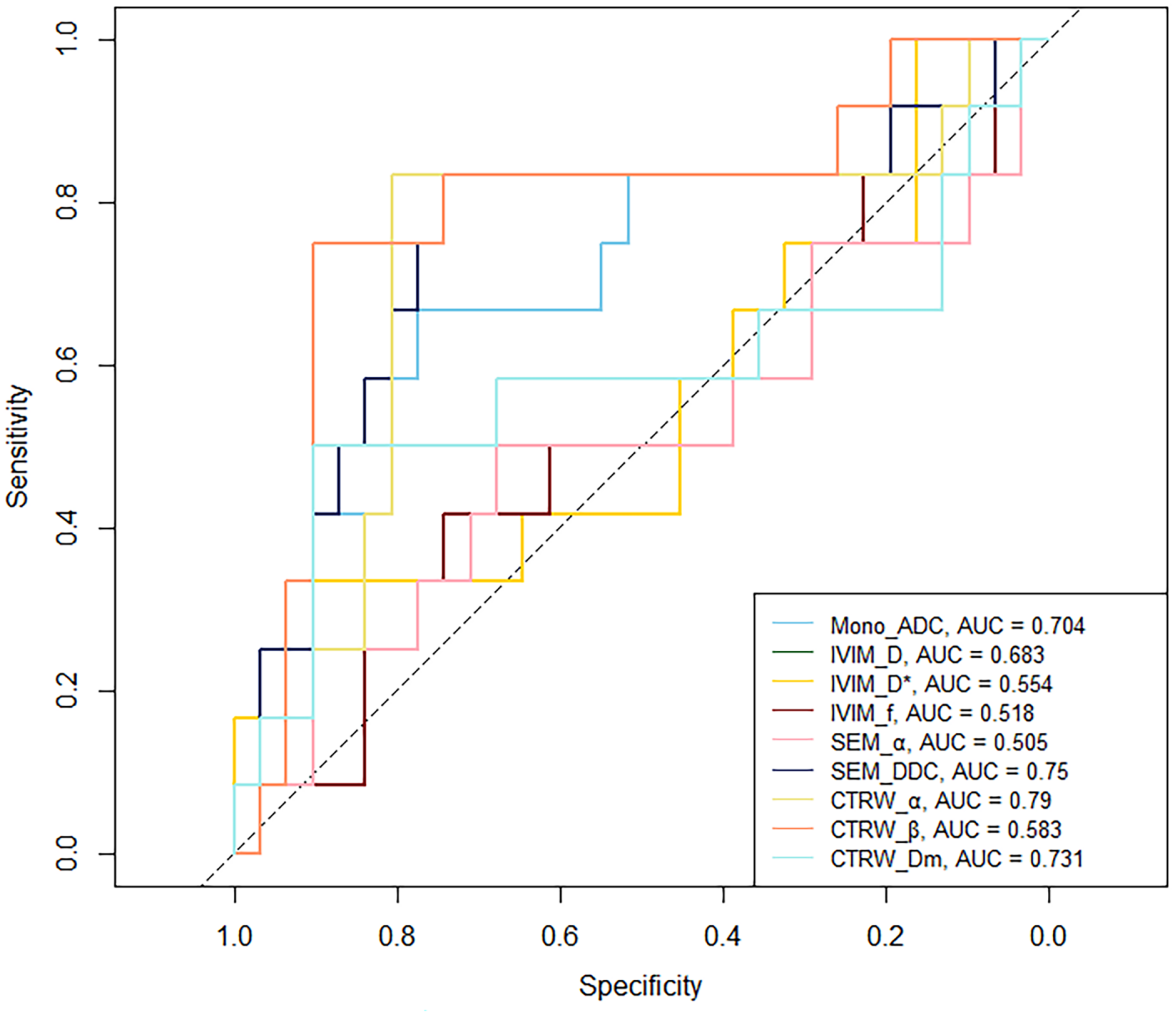
ROC curve of 1p/19q status predicting by DWI parameters.

**Figure 4:**
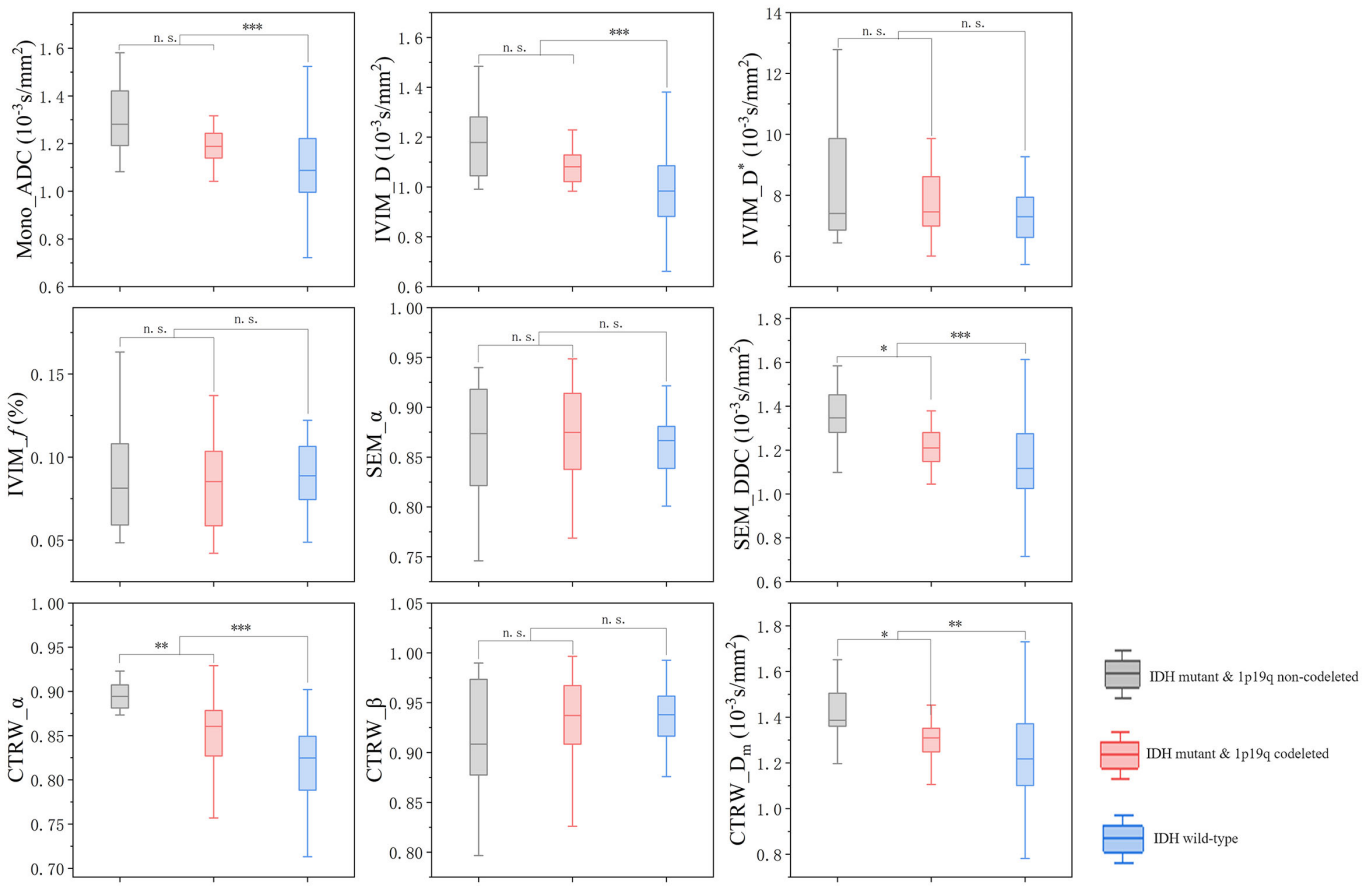
Box plot of the distribution of each parameter in three subtypes of gliomas. n.s.: no significant difference, *P = 0.01~ 0.05, **P = 0.001~ 0.01, ***P < 0.001.

In IDH-mutant gliomas, SEM_DDC, CTRW_α, and CTRW_D_m_ were significantly lower in 1p/19q codeleted gliomas compared to non-codeleted gliomas (*P* < 0.05) (**Table 4**). No significant differences were found in other diffusion parameters between the two groups (*P* > 0.05). CTRW_α had the largest effect size for predicting 1p/19q co-deletion (Cohen’s d = 0.889), as shown in **Table 4**. ROC analysis (**Table 5**) indicated that CTRW_α provided the best diagnostic performance (AUC = 0.790). At a threshold of 0.886, sensitivity was 0.750, specificity was 0.903, and accuracy was 0.860 (**Figure 3**). **Figure 1** presents representative cases of the three glioma subtypes, while **Figure 4** displays box plots of diffusion parameter distributions across the subtypes, highlighting inter-group differences.

**Table 4:** Parameter values between different 1p/19q status.

Parameter	1p/19q non-codeleted	1p/19q codeleted	*t*/*U*	*P*	Cohen’s d
Mono_ADC	1.281(1.193,1.377)	1.188(1.140,1.238)	110	0.079	0.743
IVIM_D	1.188 ± 0.159	1.089 ± 0.098	-2.014*	0.114	0.843
IVIM_D^*^	7.399(6.981,9.730)	7.453(6.991,8.541)	166	0.723	0.472
IVIM_*f*	0.087 ± 0.035	0.084 ± 0.028	-0.276*	0.882	0.094
SEM_α	0.874(0.827,0.911)	0.875(0.839,0.911)	188	0.968	-0.031
SEM_DDC	1.347(1.288,1.421)	1.21(1.155,1.272)	93	0.027	0.888
CTRW_α	0.894(0.885,0.906)	0.86(0.836,0.876)	78	0.008	0.889
CTRW_β	0.908(0.880,0.973)	0.937(0.911,0.967)	217	0.532	-0.446
CTRW_D_m_	1.387(1.364,1.466)	1.309(1.251,1.348)	100	0.043	0.640

*In line with normal distribution, an independent t-test was adopted. ADC, apparent diffusion coefficient; IVIM, intravoxel incoherent motion; D, slow diffusion coefficient; D^*^, fast diffusion coefficient; SEM, stretched exponential model; DDC, distributed diffusion coefficient; CTRW, continuous-time random walk; D_m_, anomalous diffusion coefficient.

**Table 5:** ROC curve analysis of parameters for predicting 1p/19q status.

Parameter	Threshold	*P*	AUC (95% *CI*)	Sensitivity	Specificity	Accuracy
Mono_ADC	1.250	0.016	0.704 (0.503,0.882)	0.667	0.774	0.744
IVIM_D	1.134	0.040	0.683 (0.465,0.866)	0.667	0.806	0.767
IVIM_D^*^	9.357	0.304	0.554 (0.344,0.750)	0.333	0.935	0.767
IVIM_*f*	0.100	0.469	0.508 (0.306,0.712)	0.417	0.742	0.651
SEM_α	0.853	0.481	0.505 (0.290,0.731)	0.500	0.677	0.628
SEM_DDC	1.265	0.004	0.750 (0.556,0.917)	0.833	0.742	0.767
CTRW_α	0.886	< 0.001	0.790 (0.613,0.941)	0.750	0.903	0.860
CTRW_β	0.893	0.237	0.583 (0.344,0.801)	0.500	0.903	0.791
CTRW_D_m_	1.353	0.009	0.731 (0.532,0.898)	0.833	0.806	0.814

AUC, area under the curve; CI, confidence interval; ADC, apparent diffusion coefficient; IVIM, intravoxel incoherent motion; D, slow diffusion coefficient; D^*^, fast diffusion coefficient; SEM, stretched exponential model; DDC, distributed diffusion coefficient; CTRW, continuous-time random walk; D_m_, anomalous diffusion coefficient.

### 3.4 Performance of DWI parameters in giomas with different imaging features

Our study revealed notable variations in imaging presentations between gliomas with different IDH statuses. To investigate potential underlying mechanisms, we examined associations between DWI parameter changes and radiological features and evaluating how imaging heterogeneity impacts diagnostic performance. IVIM_*f* shows a significant increase in gliomas with enhancement or cystic necrosis (**Supplementary Tables S2**, **S3**). In gliomas with enhancement, SEM_α and CTRW_α are significantly reduced (**Supplementary Table S2**). DWI parameters reveals no significant differences between gliomas with and without peritumoral edema (**Supplementary Table S4**).The cut-off values from **Table 3** was employed to evaluate the diagnostic efficacy of parameters with significant differences in gliomas with distinct molecular features. Results demonstrated that CTRW_α maintained moderate predictive accuracy (>0.7) for IDH status across all imaging manifestations (**Supplementary Table S5**).

## 4 Discussion

This study assessed the diagnostic performance of the DWI mono-exponential, IVIM, SEM, and CTRW models in predicting IDH and 1p/19q status in adult diffuse gliomas. CTRW_α exhibited the highest AUC and effect size for predicting IDH and 1p/19q status, highlighting its potential as a promising biomarker for the preoperative evaluation of these molecular characteristics in gliomas.

CTRW_α is related to the heterogeneity of time-dependent diffusion, quantifying the probability of water molecules being “trapped” or “released” as they diffuse through the complex structures and environments of biological tissues (34). A decreased CTRW_α value in IDH-wildtype gliomas implies increased subdiffusion behavior due to stronger cell-matrix adhesion, increased microstructural complexity, and a more restrictive diffusion environment. Heterogeneity information is crucial for elucidating the microstructural characteristics of tumor tissues, which are difficult to capture accurately using traditional mono-exponential models. IDH wild-type gliomas are characterized by rapid growth, and a tendency towards hemorrhage and necrosis, and their parenchyma contains cells at varying stages and/or distinct RNA transcription subtypes (48). These biological features result in more complex and heterogeneous water molecule movement in IDH wild-type glioma tissue (49) and a reduction in CTRW_α values (50). High cell density also contributes to lower α values. Wild-type IDH catalyzes the oxidative decarboxylation of isocitrate to produce α-ketoglutarate (α-KG), protecting cells from oxidative stress (6). However, mutant IDH, with neomorphic enzymatic activity, makes the oncometabolite 2-hydroxyglutarate (2-HG) (9). 2-HG reduces the proliferation rate of glioma cells, decreases angiogenesis, and results in relatively slower tumor progression and lower tissue heterogeneity within the voxel (51).

Given that the 1p/19q codeletion is primarily associated with IDH mutations (19), we evaluated the diagnostic performance of DWI parameters in preoperatively predicting the 1p/19q status in IDH-mutant gliomas. 1p/19q codeleted oligodendrogliomas are characterized by increased neovascularization and a greater propensity to involve the cortex (52), with approximately 90% of oligodendrogliomas exhibiting calcification (53). These features may lead to a more complex tumor structure and heterogeneous signal intensity, which a lower CTRW_α can exhibit. Chu et al. (49) employed diffusion kurtosis imaging and reported that oligodendrogliomas with 1p/19q co-deletion had higher mean kurtosis values compared to 1p/19q non-codeleted astrocytomas, attributing this to the increased tissue complexity in oligodendrogliomas, which supports with our findings. Additionally, oligodendrogliomas are characterized by high perfusion and rich microvascularity, which may further reduce the CTRW_α value, as the rapid signal decay at low b-values may be misinterpreted by the CTRW model as a manifestation of delayed diffusion processes.

The parameter β in the CTRW model serves as an indicator of spatial diffusion heterogeneity and is mathematically similar to the α parameter in the stretched exponential model (33). This explains the comparable performance observed between these two parameters in our study. CTRW_β and SEM_α did not exhibit significant differences across different IDH and 1p/19q genotypes in this study. Previous research (28, 43) has demonstrated that SEM_α can predict IDH status in low-grade gliomas, with IDH wild-type gliomas showing significantly lower SEM_α values than IDH-mutant gliomas. These findings are inconsistent with our results, potentially because those studies pre-classified gliomas as low-grade gliomas and glioblastomas based on the 2016 WHO classification. However, with the 2021 WHO classification update, the applicability of such study designs to current clinical practice warrants reevaluation. To better align with clinical practice, we opted not to predict glioma genetic information based on tumor grade. CTRW_β are less discriminative between IDH or 1p/19q statuses, suggesting that the primary diffusion alteration in is in temporal retention rather than spatial jumps. This supports the hypothesis that these tumors trap diffusing particles within their microenvironment rather than facilitating large-scale migratory leaps.

While CTRW_α and CTRW_β describe tissue heterogeneity from different perspectives, the exact microstructural changes in neural tissue that these parameters reflect remain unclear (54). There is a lack of direct histological evidence or relevant studies that physiologically explain the specific mechanisms by which CTRW_α and CTRW_β characterize brain tissue microstructure. Nevertheless, our findings confirm the point of Zhou et al. (55), that the CTRW model offers additional and relatively independent heterogeneity parameters compared to the SEM model, which helps overcome some SEM limitations in describing tissue heterogeneity.

CTRW model provides information on the diffusion rate of water molecules through the D_m_ value. Current research (42), based on the 2021 WHO classification criteria, indicates that ADC values predict genetic status in gliomas across all grades. The highest ADC values were observed in the IDH-mutant & 1p/19q non-codeleted subgroup, followed by the IDH-mutant & 1p/19q codeleted subgroup, with the lowest values seen in IDH wild-type gliomas, consistent with our results. IDH-mutant gliomas, due to changes in downstream protein synthesis, exhibit relatively slower tumor cell proliferation (56) and lower cell density. Among IDH-mutant gliomas, the parenchymal regions of 1p/19q codeleted gliomas typically exhibit dense and abundant cell arrangements (57). Additionally, calcification, common in 1p/19q codeleted gliomas (58), impacts MRI signal acquisition. This results in locally low parameter values on diffusion maps. Although ADC in our study is lower in oligodendrogliomas than in astrocytomas, no significant difference was found (*P* = 0.079), nor was it found for IVIM_D (*P* = 0.114). This may be due to a relatively small and unbalanced sample size. SEM_DDC seems to be the optimal parameter of diffusion rate in predicting the 1p/19q codeletion status. It showed significant differences between the two groups and had a good effective size (Cohen’s d = 0.888). CTRW_D_m_ showed significant differences between groups, but with a small effect size (Cohen’s d = 0.640), meaning the value differences between subgroups are relatively close and may limit clinical applicability. Measurements of water molecular diffusion rate within the tissue are influenced by blood flow perfusion. The tissue microenvironment and cellular density result in lower diffusion rates in oligodendrogliomas than in astrocytomas. However, the higher perfusion in oligodendrogliomas (59) increases the proportion of fast diffusion components, overestimating their diffusion rate values and diminishing the differentiation between the two tumor types. SEM_DDC offers an effective modeling approach for assessing diffusion rates in 1p/19q codeleted oligodendrogliomas. Although SEM_DDC does not explicitly separate perfusion effects, it offers improved robustness over ADC by modeling signal attenuation as a continuous distribution of diffusion rates. This allows DDC to better accommodate non-Gaussian behavior and mitigate some of the confounding effects caused by perfusion-related signal at low b-values. However, like ADC, DDC remains susceptible to perfusion contamination and does not isolate perfusion from true diffusion as in the IVIM model. IVIM_D reduces the overestimation bias in ADC caused by perfusion effects, which is especially relevant in highly vascular tissues such as oligodendrogliomas. The observed difference in IVIM_D between the two groups did not reach statistical significance (p = 0.114) with a large effect size (Cohen’s d = 0.843). Considering the relatively small sample size in the present study, this result suggests a high risk of Type II error. The potential value of IVIM_D warrants further validation in larger cohorts.

IVIM_*f* and IVIM_D^*^ provide information about tissue perfusion. Our study results indicate that IVIM-derived perfusion parameters did not show significant differences when predicting glioma genotypes across all grades. Previous studies (28, 43) have shown that IVIM_*f* and IVIM_D^*^ can be used to predict IDH genotypes in 2016 WHO glioblastomas, while no significant differences were found between IDH wild-type and IDH-mutant in WHO 2016 grade II-III gliomas. However, the findings regarding these parameters show variability in the direction of effect across different studies, leading to ongoing controversy about their roles. Such inconsistency may be related to the instability and approximate nature of IVIM_*f* and IVIM_D^*^ in characterizing perfusion. Furthermore, considering the latest WHO classification system, which prioritizes genetic subtyping before histopathological grading, our approach of analyzing gliomas without grade-based stratification may have influenced the interpretation of IVIM_f and IVIM_D^*^. Specifically, this methodology could amplify the confounding factors beyond molecular phenotype, such as tumor vascularization patterns, cellular density, and hypoxia. As a result, the statistical sensitivity for detecting genotype-related differences may have been reduced in the absence of grade-based stratification. Further evaluation of the predictive value of IVIM-derived perfusion parameters for glioma molecular information in larger datasets is warranted.

Morphological MRI results indicate that IDH mutant gliomas are more frequently located in the frontal/insula lobe, IDH wild-type gliomas more frequently exhibit enhancement, central necrosis, and peritumoral edema, which is consistent with previous study (60). Such visual differences can, to some extent, assist in differentiating different molecular subtypes of gliomas. Visual image interpretation is easy to perform, provides intuitive and rapid results, has low requirements for equipment and technology, and is widely adopted in current clinical practice. In contrast, DWI quantitative analysis has problems such as complex processing procedures and the lack of complete standardization in parameter interpretation and diagnostic criteria, which impede the widespread application of related technologies. Our findings demonstrate that imaging heterogeneity can interfere with the reflection of molecular status by DWI parameters, causing fluctuations in diagnostic performance indicators and reducing predictive efficacy. In contrast-enhanced gliomas or those with cystic/necrotic components, a higher IVIM_*f* may reflect enhanced neovascularization characteristic of these lesions. The reduction in α values derived from CTRW and DDC model suggests increased histological heterogeneity within these tumors. CTRW_α maintained moderate IDH mutation prediction accuracy (>0.7) across diverse imaging presentations, indicating parameter stability. However, CTRW_α exhibited diminished diagnostic efficacy, particularly reduced sensitivity (using IDH-mutant as positive and wildtype as negative labels), in gliomas demonstrating contrast enhancement, cystic/necrosis changes, or peritumoral edema. This diagnostic bias predisposed these lesions to misclassification as IDH-wildtype tumors, potentially leading to overestimation of clinical malignancy. These observations underscore the necessity for expanded research focusing on radiologically homogeneous lesion cohorts, which better reflects real-world clinical decision-making scenarios (61). And, the evolving paradigm of artificial intelligence (AI)-driven diagnostic systems presents promising solutions. Machine learning or deep learning algorithms capable of integratively analyzing conventional MRI features with quantitative DWI parameters may overcome current limitations through multidimensional pattern recognition. However, the development of advanced DWI models still makes sense. Considering the safety concerns of gadolinium-based contrast agents, particularly for patients with renal impairment who are unable to undergo contrast-enhanced T1 imaging, DWI techniques could be a key solution to this issue. Besides, advancements in quantitative MRI techniques may contribute to the development of AI-based predictive models by providing additional information on tumor microstructure and hemodynamics. Their roles in radiomics modeling has been well recognized (62, 63). The integration of radiomics, automated segmentation algorithms, and multiparametric MRI features enables AI models to achieve a more comprehensive characterization of tumors from both physiological and morphological perspectives. This approach facilitates more accurate noninvasive characterization of glioma genotype (64). However, several clinical implementation challenges remain such as the prolonged scanning time required for multiparametric MRI acquisition, which may not be feasible for patients with limited tolerance or emergency conditions, and the complex post-processing steps required specialized expertise and computational resources, which may limit widespread adoption in routine clinical workflows. Moreover, the development of reliable and generalizable models requires large-scale, multicenter datasets to account for inter-patient and inter-scanner variability, thereby enhancing model robustness and clinical applicability.

In this study, we assessed the predictive efficacy of the DWI mono-exponential model, IVIM, SEM, and CTRW models for glioma IDH genotyping and 1p/19q status. We confirmed the improvement of CTRW over SEM and identified the significant potential of CTRW_α. However, our study has several limitations: 1. The sample size is relatively small and imbalanced across groups; 2. The VOIs in this study were manually delineated, which introduces subjectivity. Future studies could improve this by utilizing deep learning algorithms for automatic delineation; 3. There is currently no consensus on the optimal scanning protocols or standard post-processing methods for DWI models. To improve the generalizability of multi-b-value DWI across different research centers, it is essential to standardize and validate scanning protocols and post-processing techniques.

## 5 Conclusion

CTRW model offers valuable imaging biomarkers for developing personalized treatment plans and assessing patient prognosis. The α parameter in the CTRW model has the best diagnostic performance for predicting both glioma IDH genotype and 1p/19q status.

## Data Availability

The raw data supporting the conclusions of this article will be made available by the authors, without undue reservation.

## References

[1] Weller M van den Bent M Preusser M Le Rhun E Tonn JC Minniti G. EANO guidelines on the diagnosis and treatment of diffuse gliomas of adulthood. Nat Rev Clin Oncol. (2021) 18:170–86. doi: 10.1038/s41571-020-00447-z, PMID: 33293629 PMC7904519

[2] SongTao Q Lei Y Si G YanQing D HuiXia H XueLin Z. IDH mutations predict longer survival and response to temozolomide in secondary glioblastoma. Cancer Sci. (2012) 103:269–73. doi: 10.1111/j.1349-7006.2011.02134.x, PMID: 22034964

[3] Cairncross G Wang M Shaw E Jenkins R Brachman D Buckner J. Phase III trial of chemoradiotherapy for anaplastic oligodendroglioma: long-term results of RTOG 9402. J Clin Oncol. (2013) 31:337–43. doi: 10.1200/JCO.2012.43.2674, PMID: 23071247 PMC3732012

[4] Louis DN Perry A Wesseling P Brat DJ Cree IA Figarella-Branger D. The 2021 WHO classification of tumors of the central nervous system: a summary. Neuro Oncol. (2021) 23:1231–51. doi: 10.1093/neuonc/noab106, PMID: 34185076 PMC8328013

[5] Gritsch S Batchelor TT Gonzalez Castro LN. Diagnostic, therapeutic, and prognostic implications of the 2021 World Health Organization classification of tumors of the central nervous system. Cancer. (2022) 128:47–58. doi: 10.1002/cncr.33918, PMID: 34633681

[6] Lee SM Koh HJ Park DC Song BJ Huh TL Park JW. Cytosolic NADP(+)-dependent isocitrate dehydrogenase status modulates oxidative damage to cells. Free Radic Biol Med. (2002) 32:1185–96. doi: 10.1016/s0891-5849(02)00815-8, PMID: 12031902

[7] Parsons DW Jones S Zhang X Lin JC Leary RJ Angenendt P. An integrated genomic analysis of human glioblastoma multiforme. Science. (2008) 321:1807–12. doi: 10.1126/science.1164382, PMID: 18772396 PMC2820389

[8] Goodenberger ML Jenkins RB. Genetics of adult glioma. Cancer Genet. (2012) 205:613–21. doi: 10.1016/j.cancergen.2012.10.009, PMID: 23238284

[9] Dang L White DW Gross S Bennett BD Bittinger MA Driggers EM. Cancer-associated IDH1 mutations produce 2-hydroxyglutarate. Nature. (2009) 462:739–44. doi: 10.1038/nature08617, PMID: 19935646 PMC2818760

[10] Turcan S Rohle D Goenka A Walsh LA Fang F Yilmaz E. IDH1 mutation is sufficient to establish the glioma hypermethylator phenotype. Nature. (2012) 483:479–83. doi: 10.1038/nature10866, PMID: 22343889 PMC3351699

[11] Brennan CW Verhaak RG McKenna A Campos B Noushmehr H Salama SR. The somatic genomic landscape of glioblastoma. Cell. (2013) 155:462–77. doi: 10.1016/j.cell.2013.09.034, PMID: 24120142 PMC3910500

[12] Boots-Sprenger SH Sijben A Rijntjes J Tops BB Idema AJ Rivera AL. Significance of complete 1p/19q co-deletion, IDH1 mutation and MGMT promoter methylation in gliomas: use with caution. Mod Pathol. (2013) 26:922–9. doi: 10.1038/modpathol.2012.166, PMID: 23429602

[13] Gladson CL Prayson RA Liu WM. The pathobiology of glioma tumors. Annu Rev Pathol. (2010) 5:33–50. doi: 10.1146/annurev-pathol-121808-102109, PMID: 19737106 PMC2887670

[14] Mellinghoff IK Ellingson BM Touat M Maher E de la Fuente MI Holdhoff M. Ivosidenib in isocitrate dehydrogenase 1-mutated advanced glioma. J Clin Oncol. (2020) 38:3398–406. doi: 10.1200/JCO.19.03327, PMID: 32530764 PMC7527160

[15] Karpel-Massler G Nguyen TTT Shang E Siegelin MD. Novel IDH1-targeted glioma therapies. CNS Drugs. (2019) 33:1155–66. doi: 10.1007/s40263-019-00684-6, PMID: 31768950 PMC7027940

[16] Persico P Lorenzi E Losurdo A Dipasquale A Di Muzio A Navarria P. Precision oncology in lower-grade gliomas: promises and pitfalls of therapeutic strategies targeting IDH-mutations. Cancers (Basel). (2022) 14(5):1125. doi: 10.3390/cancers14051125, PMID: 35267433 PMC8909346

[17] Tanboon J Williams EA Louis DN. The diagnostic use of immunohistochemical surrogates for signature molecular genetic alterations in gliomas. J Neuropathol Exp Neurol. (2016) 75:4–18. doi: 10.1093/jnen/nlv009, PMID: 26671986

[18] Jackson RJ Fuller GN Abi-Said D Lang FF Gokaslan ZL Shi WM. Limitations of stereotactic biopsy in the initial management of gliomas. Neuro Oncol. (2001) 3:193–200. doi: 10.1093/neuonc/3.3.193, PMID: 11465400 PMC1920616

[19] Su C Xu S Lin D He H Chen Z Damen FC. Multi-parametric Z-spectral MRI may have a good performance for glioma stratification in clinical patients. Eur Radiol. (2022) 32:101–11. doi: 10.1007/s00330-021-08175-3, PMID: 34272981

[20] Arzanforoosh F van der Voort SR Incekara F Vincent A Van den Bent M Kros JM. Microvasculature features derived from hybrid EPI MRI in non-enhancing adult-type diffuse glioma subtypes. Cancers (Basel). (2023) 15(7):2135. doi: 10.3390/cancers15072135, PMID: 37046796 PMC10093697

[21] Gao A Zhang H Yan X Wang S Chen Q Gao E. Whole-tumor histogram analysis of multiple diffusion metrics for glioma genotyping. Radiology. (2022) 302:652–61. doi: 10.1148/radiol.210820, PMID: 34874198

[22] Kern M Auer TA Picht T Misch M Wiener E. T2 mapping of molecular subtypes of WHO grade II/III gliomas. BMC Neurol. (2020) 20:8. doi: 10.1186/s12883-019-1590-1, PMID: 31914945 PMC6947951

[23] Zhang X Tian Q Wang L Liu Y Li B Liang Z. Radiomics strategy for molecular subtype stratification of lower-grade glioma: detecting IDH and TP53 mutations based on multimodal MRI. J Magn Reson Imaging. (2018) 48:916–26. doi: 10.1002/jmri.25960, PMID: 29394005

[24] He J Ren J Niu G Liu A Wu Q Xie S. Multiparametric MR radiomics in brain glioma: models comparation to predict biomarker status. BMC Med Imaging. (2022) 22:137. doi: 10.1186/s12880-022-00865-8, PMID: 35931979 PMC9354364

[25] Bonada M Rossi LF Carone G Panico F Cofano F Fiaschi P. Deep learning for MRI segmentation and molecular subtyping in glioblastoma: critical aspects from an emerging field. Biomedicines. (2024) 12(8):1878. doi: 10.3390/biomedicines12081878, PMID: 39200342 PMC11352020

[26] Yang X Lin Y Xing Z She D Su Y Cao D. Predicting 1p/19q codeletion status using diffusion-, susceptibility-, perfusion-weighted, and conventional MRI in IDH-mutant lower-grade gliomas. Acta Radiol. (2021) 62:1657–65. doi: 10.1177/0284185120973624, PMID: 33222488

[27] Nguyen TB Melkus G Taccone M Moldovan ID Ghinda D Gotfrit R. Preoperative determination of isocitrate dehydrogenase mutation in gliomas using spectral editing MRS: A prospective study. J Magn Reson Imaging. (2021) 53:416–26. doi: 10.1002/jmri.27366, PMID: 32940938

[28] Lu J Li X Li H. Perfusion parameters derived from MRI for preoperative prediction of IDH mutation and MGMT promoter methylation status in glioblastomas. Magn Reson Imaging. (2021) 83:189–95. doi: 10.1016/j.mri.2021.09.005, PMID: 34506909

[29] Le Bihan D Breton E Lallemand D Grenier P Cabanis E Laval-Jeantet M. MR imaging of intravoxel incoherent motions: application to diffusion and perfusion in neurologic disorders. Radiology. (1986) 161:401–7. doi: 10.1148/radiology.161.2.3763909, PMID: 3763909

[30] Bennett KM Schmainda KM Bennett RT Rowe DB Lu H Hyde JS. Characterization of continuously distributed cortical water diffusion rates with a stretched-exponential model. Magn Reson Med. (2003) 50:727–34. doi: 10.1002/mrm.10581, PMID: 14523958

[31] Yu Y Liang Y. A concise continuous time random-walk diffusion model for characterization of non-exponential signal decay in magnetic resonance imaging. Magn Reson Imaging. (2023) 103:84–91. doi: 10.1016/j.mri.2023.07.007, PMID: 37451520

[32] Ingo C Magin RL Colon-Perez L Triplett W Mareci TH. On random walks and entropy in diffusion-weighted magnetic resonance imaging studies of neural tissue. Magn Reson Med. (2014) 71:617–27. doi: 10.1002/mrm.24706, PMID: 23508765 PMC4930657

[33] Karaman MM Sui Y Wang H Magin RL Li Y Zhou XJ. Differentiating low- and high-grade pediatric brain tumors using a continuous-time random-walk diffusion model at high b-values. Magn Reson Med. (2016) 76:1149–57. doi: 10.1002/mrm.26012, PMID: 26519663 PMC4852163

[34] Li C Wen Y Xie J Chen Q Dang Y Zhang H. Preoperative prediction of VETC in hepatocellular carcinoma using non-Gaussian diffusion-weighted imaging at high b values: a pilot study. Front Oncol. (2023) 13:1167209. doi: 10.3389/fonc.2023.1167209, PMID: 37305565 PMC10248416

[35] Qin Y Tang C Hu Q Yi J Yin T Ai T. Assessment of prognostic factors and molecular subtypes of breast cancer with a continuous-time random-walk MR diffusion model: using whole tumor histogram analysis. J Magn Reson Imaging. (2023) 58:93–105. doi: 10.1002/jmri.28474, PMID: 36251468

[36] Karaman MM Zhang J Xie KL Zhu W Zhou XJ. Quartile histogram assessment of glioma Malignancy using high b-value diffusion MRI with a continuous-time random-walk model. NMR Biomed. (2021) 34:e4485. doi: 10.1002/nbm.4485, PMID: 33543512

[37] Sorensen AG Buonanno FS Gonzalez RG Schwamm LH Lev MH Huang-Hellinger FR. Hyperacute stroke: evaluation with combined multisection diffusion-weighted and hemodynamically weighted echo-planar MR imaging. Radiology. (1996) 199:391–401. doi: 10.1148/radiology.199.2.8668784, PMID: 8668784

[38] Chang H Wang D Li Y Xiang S Yang YX Kong P. Evaluation of breast cancer Malignancy, prognostic factors and molecular subtypes using a continuous-time random-walk MR diffusion model. Eur J Radiol. (2023) 166:111003. doi: 10.1016/j.ejrad.2023.111003, PMID: 37506477

[39] Mao C Hu L Jiang W Qiu Y Yang Z Liu Y. Discrimination between human epidermal growth factor receptor 2 (HER2)-low-expressing and HER2-overexpressing breast cancers: a comparative study of four MRI diffusion models. Eur Radiol. (2023) 34(4):2546–59. doi: 10.1007/s00330-023-10198-x, PMID: 37672055

[40] Karaman MM Wang H Sui Y Engelhard HH Li Y Zhou XJ. A fractional motion diffusion model for grading pediatric brain tumors. NeuroImage Clin. (2016) 12:707–14. doi: 10.1016/j.nicl.2016.10.003, PMID: 27761401 PMC5065039

[41] Xu J Ren Y Zhao X Wang X Yu X Yao Z. Incorporating multiple magnetic resonance diffusion models to differentiate low- and high-grade adult gliomas: a machine learning approach. Quant Imaging Med Surg. (2022) 12:5171–83. doi: 10.21037/qims-22-145, PMID: 36330178 PMC9622457

[42] Ma X Cheng K Cheng G Li C Lyu J Lan Y. Apparent diffusion coefficient as imaging biomarker for identifying IDH mutation, 1p19q codeletion, and MGMT promoter methylation status in patients with glioma. J Magn Reson Imaging. (2023) 58:732–8. doi: 10.1002/jmri.28589, PMID: 36594577

[43] Wang X Chen XZ Shi L Dai JP. Glioma grading and IDH1 mutational status: assessment by intravoxel incoherent motion MRI. Clin Radiol. (2019) 74:651.e7–e14. doi: 10.1016/j.crad.2019.03.020, PMID: 31014573

[44] Xie S Chen L Zuo N Jiang T. DiffusionKit: A light one-stop solution for diffusion MRI data analysis. J Neurosci Methods. (2016) 273:107–19. doi: 10.1016/j.jneumeth.2016.08.011, PMID: 27568099

[45] Yang L Hu H Yang X Yan Z Shi G Yang L. Whole-tumor histogram analysis of multiple non-Gaussian diffusion models at high b values for assessing cervical cancer. Abdom Radiol (NY). (2024) 49:2513–24. doi: 10.1007/s00261-024-04486-3, PMID: 38995401

[46] Kang KM Song J Choi Y Park C Park JE Kim HS. MRI scoring systems for predicting isocitrate dehydrogenase mutation and chromosome 1p/19q codeletion in adult-type diffuse glioma lacking contrast enhancement. Radiology. (2024) 311:e233120. doi: 10.1148/radiol.233120, PMID: 38713025

[47] Song Y Zhang J Zhang YD Hou Y Yan X Wang Y. FeAture Explorer (FAE): A tool for developing and comparing radiomics models. PLoS One. (2020) 15:e0237587. doi: 10.1371/journal.pone.0237587, PMID: 32804986 PMC7431107

[48] Neftel C Laffy J Filbin MG Hara T Shore ME Rahme GJ. An integrative model of cellular states, plasticity, and genetics for glioblastoma. Cell. (2019) 178:835–49 e21. doi: 10.1016/j.cell.2019.06.024, PMID: 31327527 PMC6703186

[49] Chu JP Song YK Tian YS Qiu HS Huang XH Wang YL. Diffusion kurtosis imaging in evaluating gliomas: different region of interest selection methods on time efficiency, measurement repeatability, and diagnostic ability. Eur Radiol. (2021) 31:729–39. doi: 10.1007/s00330-020-07204-x, PMID: 32857204

[50] Xie Y Li S Shen N Gan T Zhang S Liu WV. Assessment of isocitrate dehydrogenase 1 genotype and cell proliferation in gliomas using multiple diffusion magnetic resonance imaging. Front Neurosci. (2021) 15:783361. doi: 10.3389/fnins.2021.783361, PMID: 34880724 PMC8645648

[51] Bralten LB Kloosterhof NK Balvers R Sacchetti A Lapre L Lamfers M. IDH1 R132H decreases proliferation of glioma cell lines *in vitro* and *in vivo* . Ann Neurol. (2011) 69:455–63. doi: 10.1002/ana.22390, PMID: 21446021

[52] Yamashita K Togao O Kikuchi K Kuga D Sangatsuda Y Fujioka Y. Cortical high-flow sign on arterial spin labeling: a novel biomarker for IDH-mutation and 1p/19q-codeletion status in diffuse gliomas without intense contrast enhancement. Neuroradiology. (2023) 65:1415–8. doi: 10.1007/s00234-023-03186-x, PMID: 37367991

[53] Smits M. Imaging of oligodendroglioma. Br J Radiol. (2016) 89:20150857. doi: 10.1259/bjr.20150857, PMID: 26849038 PMC4846213

[54] Gatto RG Ye AQ Colon-Perez L Mareci TH Lysakowski A Price SD. Detection of axonal degeneration in a mouse model of Huntington’s disease: comparison between diffusion tensor imaging and anomalous diffusion metrics. MAGMA. (2019) 32:461–71. doi: 10.1007/s10334-019-00742-6, PMID: 30771034 PMC7837609

[55] Tang L Zhou XJ. Diffusion MRI of cancer: From low to high b-values. J Magn Reson Imaging. (2019) 49:23–40. doi: 10.1002/jmri.26293, PMID: 30311988 PMC6298843

[56] Masui K Kato Y Sawada T Mischel PS Shibata N. Molecular and genetic determinants of glioma cell invasion. Int J Mol Sci. (2017) 18(12):2609. doi: 10.3390/ijms18122609, PMID: 29207533 PMC5751212

[57] Kim SH Kim H Kim TS. Clinical, histological, and immunohistochemical features predicting 1p/19q loss of heterozygosity in oligodendroglial tumors. Acta Neuropathol. (2005) 110:27–38. doi: 10.1007/s00401-005-1020-x, PMID: 15920661

[58] Zhao K Sun G Wang Q Xue Z Liu G Xia Y. The diagnostic value of conventional MRI and CT features in the identification of the IDH1-mutant and 1p/19q co-deletion in WHO grade II gliomas. Acad Radiol. (2021) 28:e189–e98. doi: 10.1016/j.acra.2020.03.008, PMID: 32359929

[59] Latysheva A Emblem KE Brandal P Vik-Mo EO Pahnke J Roysland K. Dynamic susceptibility contrast and diffusion MR imaging identify oligodendroglioma as defined by the 2016 WHO classification for brain tumors: histogram analysis approach. Neuroradiology. (2019) 61:545–55. doi: 10.1007/s00234-019-02173-5, PMID: 30712139

[60] Delfanti RL Piccioni DE Handwerker J Bahrami N Krishnan A Karunamuni R. Imaging correlates for the 2016 update on WHO classification of grade II/III gliomas: implications for IDH, 1p/19q and ATRX status. J Neurooncol. (2017) 135:601–9. doi: 10.1007/s11060-017-2613-7, PMID: 28871469 PMC5700844

[61] Zhao K Gao A Gao E Qi J Chen T Zhao G. Multiple diffusion metrics in differentiating solid glioma from brain inflammation. Front Neurosci. (2023) 17:1320296. doi: 10.3389/fnins.2023.1320296, PMID: 38352939 PMC10861663

[62] Foltyn-Dumitru M Schell M Sahm F Kessler T Wick W Bendszus M. Advancing noninvasive glioma classification with diffusion radiomics: Exploring the impact of signal intensity normalization. Neurooncol Adv. (2024) 6:vdae043. doi: 10.1093/noajnl/vdae043, PMID: 38596719 PMC11003539

[63] Pei D Guan F Hong X Liu Z Wang W Qiu Y. Radiomic features from dynamic susceptibility contrast perfusion-weighted imaging improve the three-class prediction of molecular subtypes in patients with adult diffuse gliomas. Eur Radiol. (2023) 33:3455–66. doi: 10.1007/s00330-023-09459-6, PMID: 36853347

[64] Yuan J Siakallis L Li HB Brandner S Zhang J Li C. Structural- and DTI- MRI enable automated prediction of IDH Mutation Status in CNS WHO Grade 2–4 glioma patients: a deep Radiomics Approach. BMC Med Imaging. (2024) 24:104. doi: 10.1186/s12880-024-01274-9, PMID: 38702613 PMC11067215

