# Supplementary material for: Predicting IDH and 1p/19q molecular status of gliomas with multi-b values DWI
Source: Front Oncol. 2025 Jul 30;15:1551023. doi: 10.3389/fonc.2025.1551023 (PMC12344946; doi:10.3389/fonc.2025.1551023)
Supplement: Supplementary file 1 [file Table1.docx]

**Table S1** Intraclass correlation coefficients (ICCs) and confidence intervals for DWI parameters

|  | ICC (95% CI) |
| --- | --- |
| Mono_ADC | 0.992(0.988, 0.995) |
| IVIM_D | 0.988(0.982, 0.992) |
| IVIM_D^*^ | 0.971(0.956, 0.980) |
| IVIM_f | 0.987(0.981, 0.992) |
| SEM_α | 0.961(0.941, 0.974) |
| SEM_DDC | 0.993(0.989, 0.995) |
| CTRW_α | 0.918(0.880, 0.945) |
| CTRW_β | 0.943(0.916, 0.962) |
| CTRW_D_m_ | 0.996(0.993, 0.997) |

ICC: intraclass correlation coefficients; CI: confidence intervals

**Table S2** DWI parameter comparison based on presence of contrast enhancement

|  | Absence | Presence | *t*/*U* | *P* |
| --- | --- | --- | --- | --- |
| Mono_ADC | 1.169 ± 0.152 | 1.161 ± 0.211 | 0.195* | 0.946 |
| IVIM_D | 1.064(1.010,1.124) | 0.997(0.924,1.153) | 1124 | 0.425 |
| IVIM_D^*^ | 7.345(6.676,8.694) | 7.453(6.709,8.044) | 1007 | 0.946 |
| IVIM_*f* | 0.068(0.059,0.086) | 0.092(0.078,0.110) | 511 | 0.001 |
| SEM_α | 0.894(0.868,0.907) | 0.857(0.829,0.880) | 1380 | 0.003 |
| SEM_DDC | 1.197 ± 0.159 | 1.193 ± 0.221 | 0.068* | 0.946 |
| CTRW_α | 0.861 ± 0.040 | 0.821 ± 0.056 | 3.458 | 0.003 |
| CTRW_β | 0.956(0.929,0.97) | 0.931(0.908,0.954) | 1218 | 0.117 |
| CTRW_D_m_ | 1.272 ± 0.152 | 1.292 ± 0.222 | -0.448* | 0.946 |

* In line with normal distribution, an independent t-test was adopted. ADC = apparent diffusion coefficient, IVIM = intravoxel incoherent motion, D = slow diffusion coefficient, D^*^ = fast diffusion coefficient, SEM = stretched exponential model, DDC = distributed diffusion coefficient, CTRW = continuous-time random walk, D_m_ = anomalous diffusion coefficient

**Table S3** DWI parameter comparison based on presence of cystic/necrosis changes

|  | Absence | Presence | *t*/*U* | *P* |
| --- | --- | --- | --- | --- |
| Mono_ADC | 1.132 ± 0.174 | 1.174 ± 0.199 | -0.898* | 0.439 |
| IVIM_D | 1.038 ± 0.166 | 1.052 ± 0.175 | -0.346* | 0.730 |
| IVIM_D^*^ | 7.022(6.509,8.525) | 7.465(6.950,8.063) | 728 | 0.439 |
| IVIM_*f* | 0.069(0.056,0.080) | 0.091(0.077,0.108) | 419 | 0.002 |
| SEM_α | 0.892(0.862,0.914) | 0.862(0.832,0.884) | 1131 | 0.036 |
| SEM_DDC | 1.153 ± 0.18 | 1.208 ± 0.209 | -1.116* | 0.401 |
| CTRW_α | 0.846(0.822,0.880) | 0.836(0.792,0.867) | 971 | 0.391 |
| CTRW_β | 0.96(0.932,0.971) | 0.931(0.908,0.956) | 1099 | 0.054 |
| CTRW_D_m_ | 1.236 ± 0.172 | 1.302 ± 0.209 | -1.356* | 0.391 |

* In line with normal distribution, an independent t-test was adopted. ADC = apparent diffusion coefficient, IVIM = intravoxel incoherent motion, D = slow diffusion coefficient, D^*^ = fast diffusion coefficient, SEM = stretched exponential model, DDC = distributed diffusion coefficient, CTRW = continuous-time random walk, D_m_ = anomalous diffusion coefficient

**Table S4** DWI parameter comparison based on presence of peritumoral edema

|  | Absence | Presence | *t*/*U* | *P* |
| --- | --- | --- | --- | --- |
| Mono_ADC | 1.152 ± 0.245 | 1.169 ± 0.168 | -0.396* | 0.865 |
| IVIM_D | 1.058(0.895,1.137) | 1.015(0.970,1.133) | 994 | 0.865 |
| IVIM_D^*^ | 7.365(6.734,8.721) | 7.417(6.651,7.982) | 1013 | 0.865 |
| IVIM_*f* | 0.079 ± 0.024 | 0.091 ± 0.024 | -2.186* | 0.153 |
| SEM_α | 0.872(0.847,0.902) | 0.868(0.829,0.892) | 1075 | 0.865 |
| SEM_DDC | 1.182 ± 0.257 | 1.2 ± 0.176 | -0.332* | 0.865 |
| CTRW_α | 0.860(0.831,0.883) | 0.830(0.802,0.866) | 1219 | 0.153 |
| CTRW_β | 0.937(0.908,0.967) | 0.938(0.912,0.961) | 959 | 0.990 |
| CTRW_D_m_ | 1.259 ± 0.255 | 1.297 ± 0.175 | -0.839* | 0.865 |

* In line with normal distribution, an independent t-test was adopted. ADC = apparent diffusion coefficient, IVIM = intravoxel incoherent motion, D = slow diffusion coefficient, D^*^ = fast diffusion coefficient, SEM = stretched exponential model, DDC = distributed diffusion coefficient, CTRW = continuous-time random walk, D_m_ = anomalous diffusion coefficient

**Table S5** Parameter efficacy for IDH prediction in gliomas stratified by radiological features

|  | Sensitivity | Specificity | Accuracy |
| --- | --- | --- | --- |
| Mono_ADC |  |  |  |
| -Cystic/Necrosis(Y/N) | 0.893/0.800 | 0.659/0.750 | 0.750/0.783 |
| -Edema (Y/N) | 0.738/0.895 | 0.944/0.900 | 0.659/0.896 |
| -Enhancement (Y/N) | 0.944/0.800 | 0.660/0.800 | 0.738/0.800 |
| IVIM_D |  |  |  |
| -Cystic/Necrosis(Y/N) | 0.857/0.933 | 0.636/0.75 | 0.722/0.870 |
| -Edema (Y/N) | 0.791/1.000 | 0.595/0.900 | 0.667/0.965 |
| -Enhancement (Y/N) | 0.833/0.920 | 0.638/0.800 | 0.692/0.900 |
| SEM_DDC |  |  |  |
| -Cystic/Necrosis(Y/N) | 0.929/0.867 | 0.545/0.750 | 0.694/0.826 |
| -Edema (Y/N) | 0.833/1.000 | 0.500/0.900 | 0.621/0.965 |
| -Enhancement (Y/N) | 0.944/0.880 | 0.553/0.800 | 0.662/0.867 |
| CTRW_α |  |  |  |
| -Cystic/Necrosis(Y/N) | 0.536/0.733 | 0.818/1.000 | 0.708/0.826 |
| -Edema (Y/N) | 0.500/0.734 | 0.833/0.900 | 0.712/0.793 |
| -Enhancement (Y/N) | 0.440/0.720 | 0.830/1.000 | 0.723/0.767 |
| CTRW_D_m_ |  |  |  |
| -Cystic/Necrosis(Y/N) | 0.821/0.733 | 0.568/0.750 | 0.667/0.739 |
| -Edema (Y/N) | 0.791/0.789 | 0.524/0.900 | 0.621/0.828 |
| -Enhancement (Y/N) | 0.944/0.680 | 0.574/0.700 | 0.677/0.800 |

ADC = apparent diffusion coefficient, Y = Yes, N= No, IVIM = intravoxel incoherent motion, D = slow diffusion coefficient, SEM = stretched exponential model, DDC = distributed diffusion coefficient, CTRW = continuous-time random walk, D_m_ = anomalous diffusion coefficient
